# Genetic Variants of *CD209* Associated with Kawasaki Disease Susceptibility

**DOI:** 10.1371/journal.pone.0105236

**Published:** 2014-08-22

**Authors:** Ho-Chang Kuo, Ying-Hsien Huang, Shu-Chen Chien, Hong-Ren Yu, Kai-Sheng Hsieh, Yu-Wen Hsu, Wei-Chiao Chang

**Affiliations:** 1 Department of Pediatrics and Kawasaki Disease Center, Kaohsiung Chang Gung Memorial Hospital and Chang Gung University College of Medicine, Kaohsiung, Taiwan; 2 Department of Clinical Pharmacy, School of Pharmacy, Taipei Medical University, Taipei, Taiwan; 3 Master Program for Clinical Pharmacogenomics and Pharmacoproteomics, School of Pharmacy, Taipei Medical University, Taipei, Taiwan; 4 Department of Pharmacy, Taipei Medical University Hospital, Taipei, Taiwan; 5 Cancer Center, Kaohsiung Medical University Hospital, Kaohsiung Medical University, Kaohsiung, Taiwan; 6 Graduate Institute of Clinical Medicine, College of Medicine, Kaohsiung Medical University, Kaohsiung, Taiwan; National Central University, Taiwan

## Abstract

**Background:**

Kawasaki disease (KD) is a systemic vasculitis with unknown etiology mainly affecting children in Asian countries. Dendritic cell-specific intercellular adhesion molecule-3 grabbing non-integrin (DC-SIGN, CD209) in humans was showed to trigger an anti-inflammatory cascade and associated with KD susceptibility. This study was conducted to investigate the association between genetic polymorphisms of CD209 and the risk KD.

**Methods:**

A total of 948 subjects (381 KD and 567 controls) were recruited. Nine tagging SNPs (rs8112310, rs4804800, rs11465421, rs1544766, rs4804801, rs2287886, rs735239, rs735240, rs4804804) were selected for TaqMan allelic discrimination assay. Clinical phenotypes, coronary artery lesions (CAL) and intravenous immunoglobulin (IVIG) treatment outcomes were collected for analysis.

**Results:**

Significant associations were found between *CD209* polymorphisms (rs4804800, rs2287886, rs735240) and the risk of KD. Haplotype analysis for *CD209* polymorphisms showed that A/A/G haplotype (*P* = 0.0002, OR = 1.61) and G/A/G haplotype (*P* = 0.0365, OR = 1.52) had higher risk of KD as compared with G/G/A haplotype in rs2287886/rs735239/rs735240 pairwise allele analysis. There were no significant association in KD with regards to CAL formation and IVIG treatment responses.

**Conclusion:**

CD209 polymorphisms were responsible for the susceptibility of KD, but not CAL formation and IVIG treatment responsiveness.

## Introduction

Kawasaki disease (KD) is a systemic vasculitis which was first reported by Dr. Kawasaki in 1974 in English from Japan [1]. It mainly affects children less than 5-years-old world widely, especially in Asia. Japan, Korea and Taiwan have the highest incidence of KD worldwide from 66-234/100,000 children less than 5 years old [2], [3], [4]. The clinical characteristics and diagnosis criteria of KD include a prolonged fever (more than 5 days), bilateral non-purulent conjunctivitis, diffuse mucosal inflammation of oral cavity with strawberry tongue and fissure lips, polymorphous skin rashes over body surface, indurative angioedema of the hands and feet followed by desquamation in the sub-acute stage, and lymphadenopathy over neck [3], [5]. Fever for more than 5 days with 4 of the 5 diagnostic criteria matches the diagnosis of KD [6]. The most common sequel of KD is coronary artery lesions (CAL) formation [7], [8]. KD has become the most common cause of acquired heart disease in children of developed countries. The cause of KD is still unclear. Both genetic and environmental factors are considered to be important factors of KD. High dose intravenous immunoglobulin (IVIG) with aspirin is considered to be an effective treatment for KD [8]. The pharmacological mechanism of IVIG also remains unclear. The potential mechanisms of IVIG action include modulation of cytokine production, suppression of antibody synthesis and immune regulation [8]. IVIG therapy itself has greatly decreased the rate of aneurysms; however, some patients are unresponsive to the initial IVIG treatment. The incidence rate of IVIG resistance varies from 9.4–23% between countries [6].

IVIG is used to treat a wide range of autoimmune or immune related diseases. The immunosuppressive effects of IVIG are, in part, attributed to terminal α2,6-linked sialic acid residues on the N-linked glycans of the IgG Fc (fragment crystallizable) domain. α2,6-linked sialylated IgG was reported to interact with dendritic cell-specific intercellular adhesion molecule-3 grabbing non-integrin (*DC-SIGN, CD209*), and trigger an anti-inflammatory cascade that promotes the up regulation of inhibitory FcγRs on macrophages [9]. CD209 is a dendritic cell (DC)-specific C-type lectin superfamily receptor that has functions of pattern recognition receptor in the innate response to infection, DC migration, and the initial steps of T cell activation [10]. Several lines of evidence indicated the association between CD209 and infectious diseases, such as dengue fever, tuberculosis and AIDS [11], [12], [13], [14]. Thus, CD209 may be important in the anti-inflammatory functions of IVIG. Yu et al. showed CD209 (rs4804803) promoter variants have effects on susceptibility to KD, but not IVIG treatment response (15). Portman et al. revealed that Asians with the major allele “A” in rs2287886 of CD209 were more likely to be IVIG non-responders, but this allele is a minor allele in Hispanic and Caucasian ethnic groups [15], [16]. In this study, we hypothesized that CD209 may be involved in the susceptibility to KD, CAL formation and IVIG treatment response in KD patients.

## Materials and Methods

### Subjects

All KD patients were children who fulfilled the diagnostic criteria for KD and were admitted between 2000 and 2012 as described in our previous studies [7], [17], [18], [19]. This study was approved by the Institutional Review Board of Chang Gung Memorial Hospital (97-0029B). The IRB approved this consent procedure. Blood samples were collected after written informed consent was obtained from parents or guardians. The participant consent was recorded by decode method. We excluded patients who did not meet the diagnostic criteria for KD. CAL was defined as the internal diameter of the coronary artery greater than 3 mm (4 mm, if the subject was more than 5 year-old) or the internal diameter of a segment being at least 1.5 times than adjacent segment by echocardiogram [20], [21], [22]. IVIG responsiveness was defined as fever subside 48 hours after the completion of IVIG treatment and no fever (temperature, >38°C) recurrence for at least 7 days with marked improvement of inflammatory signs [18], [23]. A total of 567 control subjects (without any history of Kawasaki disease) were recruited from patients of outpatient department who volunteered to participate in our study while receiving an allergen test and with a negative allergen test result.

### DNA extraction

DNA was extracted by treating 0.5% SDS lysis buffer, and then adding protease K (1 mg/ml) for digestion of nuclear protein for 4 h at 60°C. After using Gentra extraction kit and followed by 70% alcohol precipitation, the total DNA was harvested.

### Genotyping

Utilizing the Han Chinese in Beijing as a reference population sample from the haplotype map database (http://www.hapmap.org), we selected the nine tagging SNPs of *CD209* (rs8112310, rs4804800, rs11465421, rs1544766, rs4804801, rs2287886, rs735239, rs735240, and rs4804804) with a minimum allele frequency of greater than 1% in the Beijing Han Chinese population. There are 4 SNPs (rs4804800, rs11465421, rs1544766, rs4804801) located on 3′ UTR, and 5 SNPs (rs8112310, rs2287886, rs735239, rs735240, rs4804804) near 5′ UTR. Genotyping was performed by using TaqMan Allelic Discrimination assay, and the polymerase chain reaction (PCR) was accomplished by using ABI StepOnePlus Thermal Cycler. Followed up in PCR, the fluorescence was detected and analyzed through the System SDS software version 2.2.2.

### Statistical analysis

All statistical analysis was performed by using JMP 9.0 for windows. The genotypes and allele frequencies associated with the susceptibility of KD and disease outcomes (CAL and IVIG treatment response) were analysis by χ^2^ test. Hardy-Weinberg equilibrium was also performed by the χ^2^ test with 1 degree of freedom. Linkage disequilibrium (LD) was assessed for haplotype blocks were defined using the default setting of the Haploview software 4.1.

## Results

### Association between *CD209* polymorphisms and susceptibility of Kawasaki disease

A total of 948 subjects (381 cases and 567 controls) were recruited in this study. The basal characteristics of KD patients and control subjects are shown in Table 1. Of the 381 KD patients, 126 (33.1%) patients had coronary artery lesion (CAL), and 49 (12.9%) patients suffered from persistent fever after they treated with IVIG. As shown in Table 2, Three SNPs (rs4804800, rs2287886, and rs735240) of *CD209* showed significance with regards to susceptibility of KD. The GG genotype of SNP rs4804800 had 1.60-fold increased risk compared with AG and AA genotypes of KD (*P* = 0.0336, OR (95% CI)  = 1.60 (1.04–2.46)). The variants of rs2287886 and rs735240 revealed protective effects, the GG genotype of rs2287886 and AA genotype of rs735240 were associated with a lower risk of KD (Table 2). However, all significances disappeared if we performed Bonfferoni correction (*P*<0.0055).

**Table 1:** Basal characteristics of patients with Kawasaki disease and normal controls.

Characteristics	Patients with KD	Normal Control
	N = 381	N = 567
Male gender, No. (%)	247 (66.8%)	314 (56.2%)
Mean (SD) age (years)	1.7±1.6	5.7±4.9
Age range (years)	0–11	0–51
CAL formation	126 (33.1%)	
IVIG resistance	49 (12.9%)	

CAL: coronary artery lesions; IVIG: intravenous immunoglobulin; SD: standard deviation.

**Table 2:** Genotype and allele frequencies of the *CD209* gene in controls and patients with Kawasaki disease.

	Genotype	Case (%) (n = 381)	Control (%) (n = 567)	MAF		*P* Value^a^	OR (95% CI)^b^
				Case	Control		
rs8112310	AA	51 (13.7)	66 (12.2)	0.361	0.359	0.5292	1.13 (0.77–1.68)
	AT	168 (44.9)	256 (47.4)				
	TT	155 (41.4)	218 (40.4)				
rs4804800	GG	46 (12.8)	45 (8.4)	0.336	0.309	**0.0336** *	**1.60 (1.04–2.46)**
	AG	149 (41.5)	240 (45.0)				
	AA	164 (45.7)	249 (46.6)				
rs11465421	TT	19 (5.4)	26 (6.0)	0.248	0.260	0.7292	0.90 (0.49–1.65)
	GT	136 (38.8)	174 (40.1)				
	GG	196 (55.8)	234 (53.9)				
rs1544766	GG	33 (8.8)	37 (6.7)	0.294	0.279	0.2404	1.34 (0.82–2.18)
	AG	154 (41.2)	232 (42.3)				
	AA	187 (50.0)	280 (51.0)				
rs4804801	AA	54 (15.2)	65 (14.6)	0.399	0.386	0.8011	1.05 (0.71–1.56)
	AT	175 (49.3)	214 (48.0)				
	TT	126 (35.5)	167 (37.4)				
rs2287886	GG	22(6.0)	56 (10.9)	0.296	0.338	0.0118*	0.52 (0.32–0.87)
	AG	174(47.3)	237 (45.9)				
	AA	172(46.7)	223 (43.2)				
rs735239	GG	9 (2.7)	10 (3.0)	0.160	0.193	0.8421	0.91 (0.37–2.27)
	AG	86 (26.4)	108 (32.6)				
	AA	231 (70.9)	213 (64.4)				
rs735240	AA	14 (3.9)	37 (7.3)	0.215	0.265	0.0337*	0.51 (0.27–0.95)
	AG	128 (35.2)	195 (38.4)				
	GG	221 (60.9)	276 (54.3)				
rs4804804	AA	77 (22.9)	87 (20.7)	0.476	0.429	0.4550	0.80 (0.56–1.14)
	AG	166 (49.4)	187 (44.4)				
	GG	93 (27.7)	147 (34.9)				

*Significant (*P*<0.05) values are in bold.

a *P* values are calculated using the Pearson's x^2^ test for the recessive model.

b ORs are for the recessive model (minor allele homozygotes versus heterozygotes and major allele homozygotes).

### *CD209* polymorphisms had no association with CAL and IVIG treatment responsiveness

The related complications and IVIG treatment responses of KD were also examined in this study. Thus, we tested the relationship between *CD209* genetic polymorphisms and CAL formation. As shown in Table 3, none of *CD209* polymorphisms significantly associated with CAL formation. In addition, we didn't find any association between the genetic variants of *CD209* and the outcomes of IVIG treatment (Table 4).

**Table 3:** Genotype and allele frequencies of *CD209* gene in patients having Kawasaki disease with or without coronary artery lesion formation.

	Genotype	CAL (%) (n = 126)	Without (%) (n = 252)	MAF		*P* Value^a^	OR (95% CI)^b^
				CAL	Without		
rs8112310	AA	16 (13.0)	35 (14.1)	0.341	0.371	0.7711	0.91 (0.48–1.72)
	AT	52 (42.3)	114 (46.0)				
	TT	55 (44.7)	99 (39.9)				
rs4804800	GG	15 (12.6)	31 (13.1)	0.315	0.346	0.8997	0.96 (0.50–1.85)
	AG	45 (37.8)	102 (43.0)				
	AA	59 (49.6)	104 (43.9)				
rs11465421	TT	4 (3.4)	15 (6.6)	0.252	0.247	0.2142	0.50 (0.16–1.50)
	GT	52 (43.7)	83 (36.2)				
	GG	63 (52.9)	131 (57.2)				
rs1544766	GG	10 (8.1)	23 (9.3)	0.285	0.298	0.7155	0.87 (0.40–1.88)
	AG	50 (40.7)	102 (41.1)				
	AA	63 (51.2)	123 (49.6)				
rs4804801	AA	16 (13.8)	37 (15.7)	0.371	0.411	0.6421	0.86 (0.46–1.62)
	AT	54 (46.5)	120 (50.8)				
	TT	46 (39.7)	79 (33.5)				
rs2287886	GG	4 (3.3)	18 (7.4)	0.314	0.287	0.1239	0.43 (0.15–1.26)
	AG	68 (56.2)	104 (42.6)				
	AA	49 (40.5)	122 (50.0)				
rs735239	GG	2 (1.9)	7 (3.2)	0.175	0.151	0.4962	0.58 (0.12–2.79)
	AG	33 (31.1)	52 (23.7)				
	AA	71 (67.0)	159 (72.9)				
rs735240	AA	2 (1.67)	12 (5.0)	0.217	0.213	0.1230	0.32 (0.08–1.36)
	AG	48 (40.0)	78 (32.5)				
	GG	70 (58.3)	150 (62.5)				
rs4804804	AA	24 (22.6)	53 (23.2)	0.500	0.469	0.9029	0.97 (0.56–1.67)
	AG	58 (54.8)	108 (47.4)				
	GG	24 (22.6)	67 (29.4)				

*Significant (*P*<0.05) values are in bold.

a *P* values are calculated using the Pearson's x^2^ test for the recessive model.

b ORs are for the recessive model (minor allele homozygotes versus heterozygotes and major allele homozygotes).

**Table 4:** Genotype and allele frequencies of the *CD209 *gene in patients with Kawasaki disease responding or not responding to intravenous immunoglobulin treatment.

	Genotype	Resistant (%) (n = 49)	Responsive (%) (n = 326)	MAF		*P* Value^a^	OR (95% CI)^b^
				Resistant	Responsive		
rs8112310	AA	9 (18.4)	41 (12.9)	0.418	0.351	0.2942	1.53 (0.69–3.36)
	AT	23 (46.9)	142 (44.5)				
	TT	17 (34.7)	136 (42.6)				
rs4804800	GG	7 (15.6)	38 (12.3)	0.367	0.330	0.5455	1.31 (0.55–3.13)
	AG	19 (42.2)	127 (41.2)				
	AA	19 (42.2)	143 (46.4)				
rs11465421	TT	1 (2.2)	18 (6.0)	0.189	0.259	0.3020	0.36 (0.05–2.52)
	GT	15 (33.3)	120 (39.9)				
	GG	29 (64.4)	163 (54.1)				
rs1544766	GG	5 (10.4)	27 (8.4)	0.323	0.289	0.6500	1.26 (0.46–3.45)
	AG	21 (43.8)	131 (40.9)				
	AA	22 (45.8)	162 (50.6)				
rs4804801	AA	11 (23.9)	42 (13.8)	0.457	0.389	0.0733	1.97 (0.94–4.13)
	AT	20 (43.5)	153 (50.1)				
	TT	15 (32.6)	110 (36.1)				
rs2287886	GG	3 (6.2)	19 (6.0)	0.271	0.299	0.9571	1.04 (0.29–3.64)
	AG	20 (41.7)	150 (47.8)				
	AA	25 (52.1)	145 (46.2)				
rs735239	GG	2 (4.7)	7 (2.5)	0.131	0.163	0.4016	1.08 (0.23–4.96)
	AG	7 (16.7)	78 (27.6)				
	AA	33 (78.6)	197 (69.9)				
rs735240	AA	2 (4.2)	12 (3.9)	0.177	0.218	0.9251	1.08 (0.23–4.96)
	AG	13 (27.1)	111 (35.9)				
	GG	33 (68.7)	186 (60.2)				
rs4804804	AA	13 (29.5)	63 (21.8)	0.511	0.472	0.2541	1.50 (0.75–3.03)
	AG	19 (43.2)	147 (50.9)				
	GG	12 (27.3)	79 (27.3)				

*Significant (*P*<0.05) values are in bold.

a *P* values are calculated using the Pearson's x^2^ test for the recessive model.

b ORs are for the recessive model (minor allele homozygotes versus heterozygotes and major allele homozygotes).

### *CD209* haplotypes associated with Kawasaki disease susceptibility

**Figure 1:**
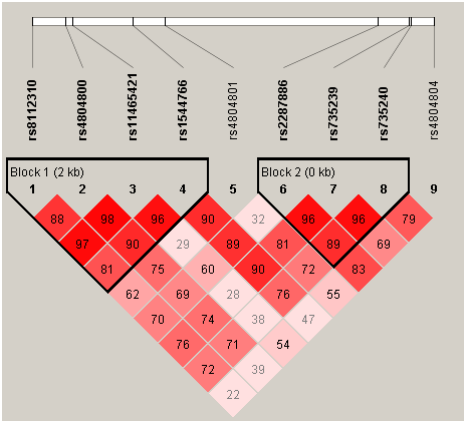
CD209 gene linkage disequilibrium and haplotype block structure in KD. The number on the cell is the LOD score of D′.

We further calculated pairwise linkage disequilibrium (LD) (Fig. 1) and analyzed haplotypes of *CD209*. The *CD209* haplotype rs8112310/rs4804800/rs11465421/rs1544766 (Block 1) had no significant association with KD susceptibility (Table 5). However, rs2287886/rs735239/rs735240 (Block 2) pairwise allele analysis showed that A/A/G haplotype (*P* = 0.0002, OR (95% CI) = 1.61 (1.25–2.08)) and G/A/G haplotype (*P* = 0.0365, OR (95% CI) = 1.52 (1.03–2.26)) had a higher risk of KD when compared with G/G/A haplotype (Table 6).

**Table 5:** Haplotype frequencies of the *CD209* gene in controls and patients with Kawasaki disease.

rs8112310/rs4804800/rs11465421/rs1544766	Case (%) (n = 381)	Control (%) (n = 567)	OR (95% CI)	*P* Value
A/G/G/G	184 (25.7)	211 (21.4)	1.23 (0.94–1.62)	0.1295
A/G/G/A	29 (4.1)	38 (3.9)	1.08 (0.64–1.82)	0.7690
T/G/G/G	12 (1.7)	15 (1.5)	1.13 (0.52–2.48)	0.7542
T/A/G/G	7 (1.0)	13 (1.3)	0.76 (0.30–1.95)	0.5701
A/A/G/A	35 (4.9)	57 (5.8)	0.87 (0.55–1.38)	0.5524
T/A/G/A	256 (35.8)	380 (38.5)	0.95 (0.75–1.22)	0.7081
T/A/T/A	185 (25.9)	262 (26.6)	Reference	

Haplotype frequency less than 1% was excluded.

**Table 6:** Haplotype frequencies of the *CD209* gene in controls and patients with Kawasaki disease.

rs2287886/rs735239/rs735240	Case (%) (n = 381)	Control (%) (n = 567)	OR (95% CI)	*P* Value
A/A/G	468 (67.2)	521 (59.5)	1.61 (1.25–2.08)	0.0002**
A/A/A	10 (1.4)	13 (1.5)	1.38 (0.59–3.25)	0.4543
G/A/G	66 (9.5)	78 (8.9)	1.52 (1.03–2.26)	0.0365*
G/A/A	27 (3.9)	36 (4.1)	1.35 (0.78–2.33)	0.2809
G/G/A	124 (17.8)	223 (25.5)	Reference	

Haplotype frequency less than 1% was excluded.

**Significant (*P*<0.01) values are in bold.

*Significant (*P*<0.05) values are in bold.

## Discussion

IVIG therapy is used to treat a wide range of autoimmune and immune associated diseases including Kawasaki disease. The treatment effects of IVIG resulted from the terminal α2,6-linked sialic acid residues of the IgG Fc (fragment crystallizable) domain, which were known to be conjugated to the carbohydrate recognition domain (CRD) of the cell-surface lectin. Through this interaction, DC-SIGN and its murine orthologue SIGN-R1 (specific intracellular adhesion molecule-grabbing non-integrin R1) triggered down-stream expression of immunosuppressive cytokines and receptors. Consistent with this model, the anti-inflammatory effect of IVIG treatment is abolished in a murine knock-out of *SIGN-R1* and can be restored by a knock-in with human *CD209* [24].

CD209 is a transmembrane lectin receptor on dendritic cells with multiple immune modulation function [25]. *CD209* can recognize many pathogens, such as viruses (HIV-1, dengue, and measles virus) [26], [27], [28], bacteria (*Helicobacter pylori*, *Mycobacterium tuberculosis*) [29], and fungi (*Candida albicans* and *Aspergillus fumigatus*) [30] contributing to the generation of pathogen-tailored immune responses and immunosuppressive responses by the MAPK pathway in DCs [31]. The pathogen which causes KD still remains unknown. The hypothesis that [32] is that risk of KD results from an undefined infectious process in a genetically predisposed individual with a “double hit model” (32). The genetic predisposition is suggested based on clinical and epidemiologic features [8], [33]. Immune related genes including *ITPKC, CD40, BLK and FcγR2A* were reported as important genetic predisposition of KD [34], [35], [36], [37].

The polymorphism of *CD209* has been previously reported as an important factor of KD susceptibility [15]. Portman's study also showed a significant association between CD209 polymorphisms and IVIG treatment in the Asian group (N = 64 vs. 12, responsiveness vs. non-responsiveness, respectively, p = 0.04) but not in the Caucasian group (158 vs. 62), Hispanic group (55 vs. 20) and group that pooled of all of the ethnicities (277 vs. 94) [16]. In this study, we conducted to investigate 9 tagging SNPs in a Taiwanese population including 332 with IVIG responsiveness and 49 with IVIG non-responsiveness. Our results indicated that CD209 polymorphisms were responsible for the susceptibility of KD, but not IVIG treatment responsiveness. Inconsistences with regards to these results may be due to the limited case numbers, power of statistical test, and different ethnic populations.

In conclusion, our results provided evidence to support the potential role of CD209 in the susceptibility of KD in a Taiwanese population. The roles of genetic polymorphisms of CD209 in inflammatory signaling as well as KD development are still unclear. Functional studies are needed to validate these findings.

## References

[1] Kawasaki T, Kosaki F, Okawa S, Shigematsu I, Yanagawa H (1974) A new infantile acute febrile mucocutaneous lymph node syndrome (MLNS) prevailing in Japan. Pediatrics 54: 271–276. 4153258

[2] Huang WC, Huang LM, Chang IS, Chang LY, Chiang BL, et al (2009) Epidemiologic features of Kawasaki disease in Taiwan, 2003–2006. Pediatrics 123: e401–405. 19237439 10.1542/peds.2008-2187

[3] Wang CL, Wu YT, Liu CA, Kuo HC, Yang KD (2005) Kawasaki disease: infection, immunity and genetics. Pediatr Infect Dis J 24: 998–1004. 16282937 10.1097/01.inf.0000183786.70519.fa

[4] Nakamura Y, Yashiro M, Uehara R, Sadakane A, Tsuboi S, et al (2012) Epidemiologic features of Kawasaki disease in Japan: results of the 2009–2010 nationwide survey. J Epidemiol 22: 216–221. 22447211 10.2188/jea.JE20110126 PMC3798622

[5] Newburger JW, Takahashi M, Gerber MA, Gewitz MH, Tani LY, et al (2004) Diagnosis, treatment, and long-term management of Kawasaki disease: a statement for health professionals from the Committee on Rheumatic Fever, Endocarditis, and Kawasaki Disease, Council on Cardiovascular Disease in the Young, American Heart Association. Pediatrics 114: 1708–1733. 15574639 10.1542/peds.2004-2182

[6] Kuo HC, Yang KD, Chang WC, Ger LP, Hsieh KS (2012) Kawasaki disease: an update on diagnosis and treatment. Pediatr Neonatol 53: 4–11. 22348488 10.1016/j.pedneo.2011.11.003

[7] Liang CD, Kuo HC, Yang KD, Wang CL, Ko SF (2009) Coronary artery fistula associated with Kawasaki disease. Am Heart J 157: 584–588. 19249434 10.1016/j.ahj.2008.11.020

[8] Burns JC, Glode MP (2004) Kawasaki syndrome. Lancet 364: 533–544. 15302199 10.1016/S0140-6736(04)16814-1

[9] Anthony RM, Ravetch JV (2010) A novel role for the IgG Fc glycan: the anti-inflammatory activity of sialylated IgG Fcs. J Clin Immunol 30 Suppl 1S9–14. 20480216 10.1007/s10875-010-9405-6

[10] Zhou T, Chen Y, Hao L, Zhang Y (2006) DC-SIGN and immunoregulation. Cell Mol Immunol 3: 279–283. 16978536

[11] Marzi A, Gramberg T, Simmons G, Moller P, Rennekamp AJ, et al (2004) DC-SIGN and DC-SIGNR interact with the glycoprotein of Marburg virus and the S protein of severe acute respiratory syndrome coronavirus. J Virol 78: 12090–12095. 15479853 10.1128/JVI.78.21.12090-12095.2004 PMC523257

[12] Vannberg FO, Chapman SJ, Khor CC, Tosh K, Floyd S, et al (2008) CD209 genetic polymorphism and tuberculosis disease. PLoS One 3: e1388. 18167547 10.1371/journal.pone.0001388 PMC2148105

[13] Koizumi Y, Kageyama S, Fujiyama Y, Miyashita M, Lwembe R, et al (2007) RANTES -28G delays and DC-SIGN - 139C enhances AIDS progression in HIV type 1-infected Japanese hemophiliacs. AIDS Res Hum Retroviruses 23: 713–719. 17530998 10.1089/aid.2006.0225

[14] Sarkar R, Mitra D, Chakrabarti S (2013) Correction: HIV-1 Gp120 Protein Downregulates Nef Induced IL-6 Release in Immature Dentritic Cells through Interplay of DC-SIGN. PLoS One 8. 10.1371/journal.pone.0059073 PMC3598654 23554973

[15] Yu HR, Chang WP, Wang L, Lin YJ, Liang CD, et al (2012) DC-SIGN (CD209) promoter -336 A/G (rs4804803) polymorphism associated with susceptibility of Kawasaki disease. ScientificWorldJournal 2012: 634835. 22629172 10.1100/2012/634835 PMC3354554

[16] Portman MA, Wiener HW, Silva M, Shendre A, Shrestha S (2013) DC-SIGN gene promoter variants and IVIG treatment response in Kawasaki disease. Pediatr Rheumatol Online J 11: 32. 24006904 10.1186/1546-0096-11-32 PMC3847673

[17] Kuo HC, Liang CD, Yu HR, Wang CL, Lin IC, et al (2011) CTLA-4, Position 49 A/G Polymorphism Associated with Coronary Artery Lesions in Kawasaki Disease. J Clin Immunol 31(2): 240–244. 21082224 10.1007/s10875-010-9484-4

[18] Kuo HC, Liang CD, Wang CL, Yu HR, Hwang KP, et al (2010) Serum albumin level predicts initial intravenous immunoglobulin treatment failure in Kawasaki disease. Acta Paediatr 99: 1578–1583. 20491705 10.1111/j.1651-2227.2010.01875.x

[19] Yu HR, Kuo HC, Sheen JM, Wang L, Lin IC, et al (2009) A unique plasma proteomic profiling with imbalanced fibrinogen cascade in patients with Kawasaki disease. Pediatr Allergy Immunol 20: 699–707. 19170925 10.1111/j.1399-3038.2008.00844.x

[20] Shulman ST, De Inocencio J, Hirsch R (1995) Kawasaki disease. Pediatr Clin North Am 42: 1205–1222. 7567192 10.1016/s0031-3955(16)40059-3

[21] Kuo HC, Yu HR, Juo SH, Yang KD, Wang YS, et al (2011) CASP3 gene single-nucleotide polymorphism (rs72689236) and Kawasaki disease in Taiwanese children. J Hum Genet 56: 161–165. 21160486 10.1038/jhg.2010.154

[22] Kuo HC, Wang CL, Liang CD, Yu HR, Chen HH, et al (2007) Persistent monocytosis after intravenous immunoglobulin therapy correlated with the development of coronary artery lesions in patients with Kawasaki disease. J Microbiol Immunol Infect 40: 395–400. 17932598

[23] Kuo HC, Yang KD, Liang CD, Bong CN, Yu HR, et al (2007) The relationship of eosinophilia to intravenous immunoglobulin treatment failure in Kawasaki disease. Pediatr Allergy Immunol 18: 354–359. 17584314 10.1111/j.1399-3038.2007.00516.x

[24] Yu X, Vasiljevic S, Mitchell DA, Crispin M, Scanlan CN (2013) Dissecting the molecular mechanism of IVIg therapy: the interaction between serum IgG and DC-SIGN is independent of antibody glycoform or Fc domain. J Mol Biol 425: 1253–1258. 23416198 10.1016/j.jmb.2013.02.006

[25] Gringhuis SI, den Dunnen J, Litjens M, van Het Hof B, van Kooyk Y, et al (2007) C-type lectin DC-SIGN modulates Toll-like receptor signaling via Raf-1 kinase-dependent acetylation of transcription factor NF-kappaB. Immunity 26: 605–616. 17462920 10.1016/j.immuni.2007.03.012

[26] Geijtenbeek TB, Kwon DS, Torensma R, van Vliet SJ, van Duijnhoven GC, et al (2000) DC-SIGN, a dendritic cell-specific HIV-1-binding protein that enhances trans-infection of T cells. Cell 100: 587–597. 10721995 10.1016/s0092-8674(00)80694-7

[27] Pokidysheva E, Zhang Y, Battisti AJ, Bator-Kelly CM, Chipman PR, et al (2006) Cryo-EM reconstruction of dengue virus in complex with the carbohydrate recognition domain of DC-SIGN. Cell 124: 485–493. 16469696 10.1016/j.cell.2005.11.042

[28] de Witte L, Abt M, Schneider-Schaulies S, van Kooyk Y, Geijtenbeek TB (2006) Measles virus targets DC-SIGN to enhance dendritic cell infection. J Virol 80: 3477–3486. 16537615 10.1128/JVI.80.7.3477-3486.2006 PMC1440360

[29] Gringhuis SI, den Dunnen J, Litjens M, van der Vlist M, Geijtenbeek TB (2009) Carbohydrate-specific signaling through the DC-SIGN signalosome tailors immunity to Mycobacterium tuberculosis, HIV-1 and Helicobacter pylori. Nat Immunol 10: 1081–1088. 19718030 10.1038/ni.1778

[30] Cambi A, Gijzen K, de Vries JM, Torensma R, Joosten B, et al (2003) The C-type lectin DC-SIGN (CD209) is an antigen-uptake receptor for Candida albicans on dendritic cells. Eur J Immunol 33: 532–538. 12645952 10.1002/immu.200310029

[31] Mittal R, Bulgheresi S, Emami C, Prasadarao NV (2009) Enterobacter sakazakii targets DC-SIGN to induce immunosuppressive responses in dendritic cells by modulating MAPKs. J Immunol 183: 6588–6599. 19846880 10.4049/jimmunol.0902029 PMC2796599

[32] Lidar M, Lipschitz N, Langevitz P, Shoenfeld Y (2009) The infectious etiology of vasculitis. Autoimmunity 42: 432–438. 19811260 10.1080/08916930802613210

[33] Nakamura Y, Yashiro M, Uehara R, Oki I, Watanabe M, et al (2008) Epidemiologic features of Kawasaki disease in Japan: results from the nationwide survey in 2005–2006. J Epidemiol 18: 167–172. 18635901 10.2188/jea.JE2008001 PMC4771586

[34] Onouchi Y, Gunji T, Burns JC, Shimizu C, Newburger JW, et al (2008) ITPKC functional polymorphism associated with Kawasaki disease susceptibility and formation of coronary artery aneurysms. Nat Genet 40: 35–42. 18084290 10.1038/ng.2007.59 PMC2876982

[35] Onouchi Y, Ozaki K, Burns JC, Shimizu C, Terai M, et al (2012) A genome-wide association study identifies three new risk loci for Kawasaki disease. Nat Genet 44: 517–521. 22446962 10.1038/ng.2220

[36] Lee YC, Kuo HC, Chang JS, Chang LY, Huang LM, et al (2012) Two new susceptibility loci for Kawasaki disease identified through genome-wide association analysis. Nat Genet 44: 522–525. 22446961 10.1038/ng.2227

[37] Khor CC, Davila S, Breunis WB, Lee YC, Shimizu C, et al (2011) Genome-wide association study identifies FCGR2A as a susceptibility locus for Kawasaki disease. Nat Genet 43: 1241–1246. 22081228 10.1038/ng.981

